# Identifying miRNAs in multiple sclerosis gray matter lesions that correlate with atrophy measures

**DOI:** 10.1002/acn3.51365

**Published:** 2021-05-12

**Authors:** Ajai Tripathi, Ishani Pandit, Aaron Perles, Yadi Zhou, Feixiong Cheng, Ranjan Dutta

**Affiliations:** ^1^ Department of Neurosciences Cleveland Clinic Cleveland Ohio USA; ^2^ Cleveland Clinic Lerner College of Medicine Cleveland Ohio USA; ^3^ Genomic Medicine Institute Cleveland Clinic Cleveland Ohio USA

## Abstract

**Objective:**

Multiple sclerosis (MS) is an inflammatory, demyelinating and neurodegenerative disease of the central nervous system (CNS). Though MS was initially considered to be a white matter demyelinating disease, myelin loss in cortical gray matter has been reported in all disease stages. We previously identified microRNAs (miRNAs) in white matter lesions (WMLs) that are detected in serum from MS patients. However, miRNA expression profiles in gray matter lesions (GMLs) from progressive MS brains are understudied.

**Methods:**

We used a combination of global miRNAs and gene expression profiling of GMLs and independent validation using real‐time quantitative polymerase chain reaction (RT‐qPCR), immuno‐in situ hybridization, and immunohistochemistry.

**Results:**

Compared to matched myelinated gray matter (GM) regions, we identified 82 miRNAs in GMLs, of which 10 were significantly upregulated and 17 were significantly downregulated. Among these 82 miRNAs, 13 were also detected in serum and importantly were associated with brain atrophy in MS patients. The predicted target mRNAs of these miRNAs belonged to pathways associated with axonal guidance, TGF‐β signaling, and FOXO signaling. Further, using state‐of‐the‐art human protein–protein interactome network analysis, we mapped the four key GM atrophy‐associated miRNAs (hsa‐miR‐149*, hsa‐miR‐20a, hsa‐miR‐29c, and hsa‐miR‐25) to their target mRNAs that were also changed in GMLs.

**Interpretation:**

Our study identifies miRNAs altered in GMLs in progressive MS brains that correlate with atrophy measures. As these miRNAs were also detected in sera of MS patients, these could act as markers of GML demyelination in MS.

## Introduction

The chronic inflammatory demyelinating and neurodegenerative disease MS is characterized by oligodendrocyte death, myelin loss, and remyelination failure.^1^ Studies of demyelination in MS have mainly focused on myelin loss from white matter (WM) regions of central nervous system (CNS) tissue, which are easily detected by noninvasive magnetic resonance imaging (MRI) techniques.^2^ Conversely, many clinical characteristics like cognitive dysfunction and neurological disability have indicated cortical involvement associated with demyelination or secondary neurodegeneration.^3^ In addition, early cortical involvement has been found to be associated with more rapid disease progression.^4^ Unfortunately, cortical brain regions are relatively difficult to identify with conventional MRI techniques. Hence, there is a pressing need to develop other markers to reliably capture cortical MS pathology, to understand disease pathogenesis, and to determine the potential impact of disease‐modifying therapies.

MicroRNAs (miRNAs), which are small noncoding RNA molecules (19‐23nt), regulate post‐transcriptional gene expression by binding to the 3' untranslated region (UTR) of a target mRNA.^5^ miRNA expression levels in different biological samples originating from MS patients have been found to be dysregulated.^6^, ^7^, ^8^ We recently reported miRNA expression profiles of demyelinated white matter lesions (WMLs) from progressive MS brains.^9^ Of these, 101 miRNAs were differentially expressed (48 upregulated and 53 downregulated, *p* < 0.05) between normal‐appearing WM and WMLs in MS brains. In a separate study, serum miRNAs levels were correlated with brain atrophy and other MRI measurements.^10^ We used our results and those from this study to identify a unique set of miRNAs in both serum and brain lesions. Though miRNA expression from cortical demyelinated gray matter lesions (GMLs) have not been widely reported, a recent study by Fritsche et al^11^ identified a cohort of dysregulated miRNAs in type III cortical lesions. Of the seven significantly upregulated miRNAs in GMLs, five (miR‐330‐3p, miR‐4286, miR‐4488, let‐7e‐5p, and miR‐432‐5) had common a target, synaptotagmin7. However, comprehensive analyses of miRNAs in GMLs, including the pathways altered, target genes expressed, and their association with MRI measurements, are lacking.

In this current study, we therefore sought to (a) analyze the expression of miRNAs and mRNAs in GMLs, (b) identify the biological pathways altered in MS brains following cortical demyelination, and (c) identify the miRNAs in MS GMLs that correlate with atrophy measures and are detected in sera from MS patients. Collectively, our findings identify miRNAs in GMLs in MS brains that correlate with atrophy measures, which opens up the possibility of using these as potential biomarkers of cortical lesions in MS.

## Materials and methods

### Human subjects and regulatory compliance

Patient demographics for this study are listed in Table 1. All brains were collected as part of the tissue procurement program approved by the Cleveland Clinic Institutional Review Board. Brains were removed according to a rapid autopsy protocol and sliced (1 cm thick) using a guided box. Slices were either rapidly frozen for biochemical analysis or long fixed in 4% paraformaldehyde (PFA) for morphological studies.^12^

**Table 1:** MS patient demographics used in miRNA profiling, mRNA expression profiling, and RT‐qPCR validation study.

Sample#	MS types	Age (years)/sex	PMI (h)	Disease duration (years)	EDSS	Lesion type	
1	SPMS	65/F	7	19	8	NAGM	miRNAs profiling
2	SPMS	53/F	5	19	9	NAGM	
3	SPMS	52/M	6	30	9	NAGM	
4	PPMS	63/M	4	10	8	NAGM	
5	PPMS	57/F	5	15	6.5	NAGM	
6	PPMS	62/F	4	46	8	GML	
7	SPMS	46/F	5	14	8	GML	
8	SPMS	66/F	13	35	8	GML	
9	SPMS	59/F	5	38	7	GML	
10	SPMS	48/F	5	27	9	GML	
11	SPMS	45/M	3	36	7	NAGM, GML	mRNA expression profiling
12	SPMS	60/F	8	29	9	NAGM, GML	
13	SPMS	52/M	5	25	9.5	NAGM, GML	
14	PPMS	63/F	5	9	7.5	GML	
15	SPMS	53/F	6	19	9	NAGM, GML	
16	SPMS	52/M	7	30	9	NAGM, GML	
17	SPMS	97/F	14	45	7.5	NAGM, GML	
18	PPMS	70/M	10	17	6.5	GML	
19	RRMS	59/M	9	10	8.5	NAGM	RT‐qPCR validation
20	SPMS	50/F	9	17	8	NAGM, GML	
21	SPMS	77/F	6	54	9	NAGM	
22	SPMS	61/M	10	43	6.5	NAGM	
23	SPMS	67/M	11	25	8	GML	
24	PPMS	70/F	9	17	8	GML	
25	PPMS	69/F	6	20	9	GML	

Abbreviations: EDSS, Expanded Disability Status Scale; GML, gray matter lesion; NAGM, normal appearing gray matter; PMI, post mortem interval (h); PPMS, primary progressive MS; RRMS, Relapse remitting MS; SPMS, secondary progressive MS.

### Global miRNA and mRNA profiling and pathway analyses

miRNAs expression profiling of GMLs (five MS normal appearing gray matter [NAGM] and five MS demyelinated cortical lesions) was performed similarly as previously reported.^9^, ^13^ For mRNA expression profiling, eight NAGM regions (*n* = 6 MS brains) and eight GMLs (*n* = 8 MS brains) were selected for microarray gene expression analysis. Details of RNA isolation, labeling, and microarray analysis have been described previously.^14^, ^15^ Significantly dysregulated miRNAs/genes have been presented as heatmaps created with the Broad Institute Morpheus online tool (https://software.broadinstitute.org/morpheus). To identify miRNA‐associated molecular pathways, we used DIANA‐miRPath v3.0, which utilizes predicted or validated miRNA target interactions derived from DianaTarBase (http://www.microrna.gr/miRPathv3).^16^ For this analysis, we applied default parameters (*p* value threshold‐0.05 and MicroT threshold‐0.08) and the human gene database to find associated KEGG pathways.

Further, to identify enriched pathways (activated and inhibited) categories associated with differentially expressed genes (DEGs), ingenuity pathway analysis (IPA) was used. We applied g:Profiler, a functional enrichment analysis tool, to classify gene ontology (GO) enriched pathways in GML DEGs.^17^ Graphical presentations of KEGG pathways were created with GraphPad Prism8 with *p* value < 0.05.

### Comparison of GML miRNAs to brain atrophy‐associated miRNAs

Regev et al^10^ found significant clinical associations (protective/pathogenic) between serum miRNA levels and cortical MRI measurements. We compared the reported miRNAs from the Regev et al^10^ study with the current study to identify the miRNAs present in both brain tissue and sera of MS patients and correlated with cortical gray matter volume (cGMV). The study reported 43 miRNAs correlating with MRI measures in Cohort 1 and 45 in Cohort 2, with only one miRNA common between the two cohorts—hsa‐miR‐185‐5p.^10^

### Quantitative RT‐PCR

Expression levels of miRNAs were determined in a separate validation cohort consisting of seven progressive MS brain samples (Table 1). To validate miRNA expression, small RNAs were isolated from GMLs and surrounding NAGM using a miRNA isolation kit (miRNeasy Mini Kit, #217004) as per manufacturer's instructions (Qiagen Inc., USA). Isolated miRNAs were reverse‐transcribed to cDNA with a TaqMan miRNA RT Kit (Applied Biosystems, #4366596) as recommended by the supplier. The expression of selected miRNAs (Table S1) was validated using TaqMan miRNA assays (cat# 4427975). U6 snRNA (assay ID #001973) was used as endogenous controls in the reaction. TaqMan Gene Expression Assays had a polymerase chain reaction (PCR) efficiency of 100% (±10%). Each sample was run in triplicate. Delta (Δ) Ct values were used to determine relative expression changes (fold change, 2^−ΔΔCT^). All quantitative data were analyzed and are presented as mean ± standard error of the mean (mean ± sem) using Prism8 (GraphPad Software).

### In situ hybridization

In situ hybridization (ISH) was performed using a modified in situ protocol with locked nucleic acid‐modified oligonucleotide probes (Exiqon, Denmark) as previously described,^9^ with a few modifications. Briefly, well‐characterized formalin‐fixed, paraffin‐embedded (FFPE) 7‐µM‐thick sections were de‐paraffinized and rehydrated followed by treatment with proteinase K (40 ng) at 37°C/30 min and then fixed in 4% PFA. Next, phosphate‐buffered saline (PBS) washed sections were incubated in imidazole buffer, followed by incubation in EDC‐Imidazole solution for 90 minutes at room temperature. After washing the sections, a DIG‐labeled probe was hybridized to each section overnight (54°C). The next morning, sections were washed in 0.1X saline‐sodium citrate, followed by endogenous peroxidase activity blocking with 3% H_2_O_2_. Sections were then placed in blocking solution (Roche) for 1 hour and incubated in α‐DIG‐POD antibody (Roche) and 1° antibody (MBP, Abcam, cat#MAB386) overnight at room temperature. The next morning, sections were washed (PBS/Tris‐HCl/Triton buffer) and incubated with fluorescent‐tagged tyramide signal amplification (Perkin Elmer, cat# NEL741E001KT) to label the probe. After washing, sections were incubated with alexa‐594‐tagged secondary antibody (ThermoFisher Scientific) for 1 hour at room temperature. Slides were then washed in 1X PBS, fixed in filtered auto‐fluorescent eliminator regent (Millipore, cat#2160), and subjected to a series of 70% ethanol washes (6 times), with a final wash in PBS. Sections were then mounted in prolong gold antifade reagent (Invitrogen, cat#P36930) and micrographed under a fluorescent microscope (Leica DM5500 B).

### Immunohistochemistry

Immunohistochemistry was performed on fresh‐frozen and paraffin‐embedded human brain sections.^12^ Fresh‐frozen human brain sections were fixed in 4% PFA for 10 minutes at room temperature, followed by peroxidase enzyme deactivation. Human MS brain paraffin sections were de‐paraffinized and rehydrated followed by antigen retrieval (10‐mM citrate buffer, pH 6). After washing in PBS, peroxidase enzyme was deactivated using 3% H_2_O_2_ in 2% Triton X‐100 solution (in PBS) in both tissue types. Sections were blocked in 5% normal goat serum (0.3% Triton X‐100 in PBS) for an hour at room temperature, followed by overnight incubation in primary antibodies (rat anti‐proteolipid protein [PLP], 1:250, kindly provided by Dr. Wendy Macklin, University of Colorado; rabbit anti‐COL5A2—1:500, Boster Inc., rabbit anti‐RDX—1:200, Sigma; rabbit anti‐BCL2—1:200, Proteintech Inc.) at 4°C. The next morning, after washing in PBS sections were incubated in primary antibody host‐specific biotin‐tagged secondary antibody for 1 hour at room temperature, followed by Avidin‐Biotin complex staining as per the manufacturer's suggestion (Vector lab, cat#PK‐6100). Sections were washed in PBS, developed with 3,3′‐diaminobenzidine tablets (DAB, Sigma, cat#D5905) and 0.012% H_2_O_2_, dehydrated, and mounted before imaging.

### Ingenuity pathway analysis (IPA) of miRNA‐target gene signatures

To correlate miRNA regulation of gene expression in similar GMLs, datasets of miRNAs and mRNAs were queried for inverse correlations of miRNA expression and its predicted/confirmed target gene (upregulated miRNAs to downregulated target genes/downregulated miRNA expression to upregulated target genes) using IPA tools, with core analysis being performed with the default setting.

### Human interactome network analysis

To identify the associations between miRNAs and DEGs, we examined the protein–protein interactions (PPIs) of the miRNA targets and the DEGs using our state‐of‐the‐art network medicine methodologies.^18^, ^19^ Detailed descriptions can be found in our recent publications.^20^, ^21^, ^22^ Briefly, to construct a comprehensive human interactome network, five types of experimental evidence of PPIs were collected: (1) PPIs from protein three‐dimensional structures; (2) binary PPIs tested by high‐throughput yeast‐two‐hybrid (Y2H) systems; (3) kinase–substrate interactions by literature‐derived low‐throughput and high‐throughput experiments, (4) literature‐curated PPIs identified by affinity purification followed by mass spectrometry, Y2H, and by literature‐derived low‐throughput experiments; and (5) signaling networks supported by literature‐derived low‐throughput experiments. The final human protein interactome contains 351,444 PPIs among 17,706 proteins. The miRNA‐target interactome was retrieved from the miRTarBase.^23^ After filtering for functional miRNA‐target interactions, the miRNA‐target interactome contains 8157 interactions among 735 miRNAs and 2766 genes. We then extracted the PPIs among the miRNA targets and the DEGs and visualized them as networks using Gephi 0.9.2 (https://gephi.org/).

## Results

### miRNA expression profiles of demyelinated cortical MS lesions

**Figure 1:**
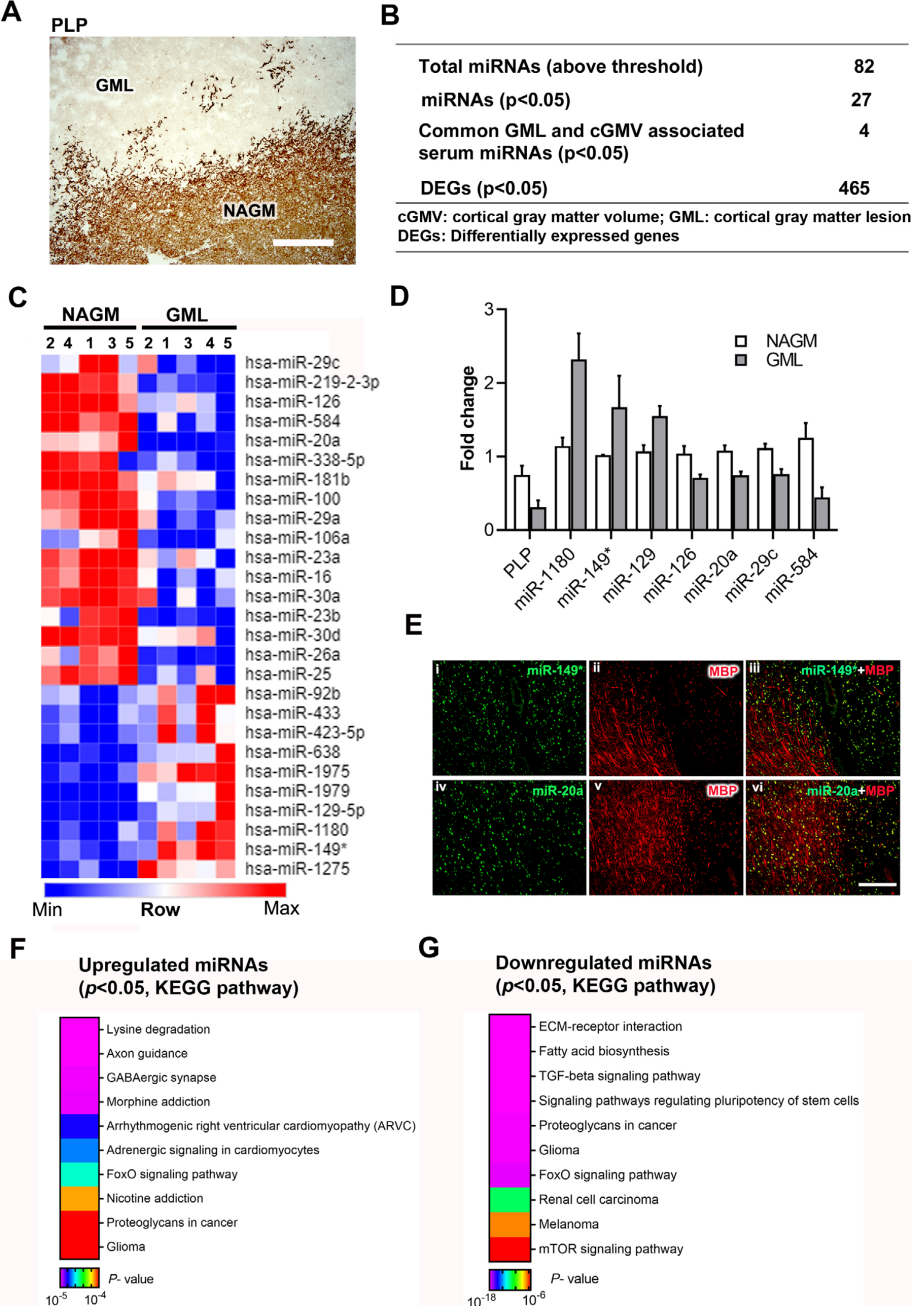
miRNAs are dysregulated in cortical demyelinated lesions. (A) Representative immunohistochemical staining of proteolipid protein (PLP) showing a demyelinated GML and surrounding NAGM from a progressive MS brain. Scale bar—200 µm. (B) Summary table of microarray miRNAs and gene expression analysis. (C) Heatmap image shows significantly upregulated and downregulated miRNAs identified in GMLs. (D) qPCR validation analysis of PLP and selected miRNA expression in NAGM and GMLs from progressive MS brains (*n* = 3–4). (E) Immuno‐in situ hybridization (immuno‐in situ) of miRNAs (green) and myelin protein (MBP, red) showing miR‐149* (i–iii) and miR‐20a (iv–vi) in myelinated and demyelinated lesioned areas of progressive MS brain. miR‐149* showed increased expression in lesioned areas, whereas miR‐20a was decreased in GMLs. GML, gray matter lesion; NAGM, normal‐appearing gray matter. (F, G) Representative heatmap image showing KEGG pathways of genes targeted by (F) upregulated and (G) downregulated miRNAs in GMLs.

Comparative analyses of miRNAs were conducted by LC Sciences, Houston, TX (Details of the array, miRNA probe sequences, and quality control measurements are listed online [at www.lcsciences.com].) using miRNA isolated from NAGM and GMLs (Type III/subpial lesions)^13^ (Fig. 1A and Table 1). Of the 496 detected miRNAs, 82 (Fig. 1B and Table S1) showed significantly higher expression over background in both NAGM and GMLs and thus were selected for further downstream analysis. Comparative analyses revealed that 27 miRNAs (33%) were significantly altered (Fig. 1B, *p* < 0.05) in GMLs. Of these 27 miRNAs, 10 were upregulated and 17 were downregulated in GMLs (Fig. 1C and Table S1). Recently, Fritsche et al^11^ found 31 significantly altered miRNAs in demyelinated cortical brain regions of MS brains (*n* = 8) compared to healthy control myelinated brain tissue (*n* = 14). Of this list, four miRNAs (miR‐1180‐3p, miR‐219‐a‐2‐3p, miR‐328‐3p, and miR‐432‐5p) were also identified in our study. Further, compared to our previous report on WML miRNAs, 78 miRNAs were common between the two lesion types, including nine significantly dysregulated miRNAs (Table 2).^9^ Interestingly, six of these nine miRNAs (miR‐1275, miR‐26a, miR‐16, miR‐23b, miR‐338‐5p, and miR129‐5p) also had similar patterns of expression between WMLs and GMLs.

**Table 2:** List of miRNAs common between cortical (GML) and white matter lesions (WML)^42^ in MS brain (miRNAs shown in bold letters have similar expression patterns between GMLs and WMLs).

miRNAs	GML/NAGM	*p* value	WML/NAWM	*p* value
hsa‐miR‐100	0.5919	0.001	1.314	0.045
hsa‐miR‐1275	1.7283	0.001	12.381	0.040
hsa‐miR‐26a	0.8272	0.004	0.596	0.003
hsa‐miR‐16	0.7162	0.004	0.641	0.008
hsa‐miR‐23b	0.7651	0.007	0.651	0.028
hsa‐miR‐25	0.8468	0.008	1.250	0.028
hsa‐miR‐338‐5p	0.5662	0.017	0.252	0.001
hsa‐miR‐30d	0.7723	0.023	1.533	0.024
hsa‐miR‐129‐5p	1.6150	0.027	2.312	0.012

To further validate the microarray findings, we selected the top four significantly upregulated and top four downregulated miRNAs (Table S1) and measured their expression levels in an independent cohort of GMLs isolated from progressive MS brains (Table 1). Using real‐time quantitative polymerase chain reaction (RT‐qPCR), we found seven miRNAs (hsa‐miR‐149*, hsa‐miR‐1180, miR‐129, hsa‐miR‐20a, hsa‐miR‐584, hsa‐miR‐126, and hsa‐miR‐29c) that showed similar expression patterns as those observed in the global microarray expression analysis (Fig. 1D). We further validated the expression of the two miRNAs, miR‐149* and miR‐20a, using immuno‐in situ (miR‐149*, Fig. 1F, i–iii and miR‐20a, iv–vi) showing predominantly neuronal expression of the miRNAs in demyelinating cortical lesions.

Searching through miRNA target gene‐enriched molecular pathways (DIANA‐miRPath v.3),^16^ several diverse pathways were found to be targeted by the 82 miRNAs, such as extracellular matrix (ECM)‐receptor interaction, axon guidance, adherens junction, and hippocampal signaling pathway (Table S2). In addition, KEGG (Kyoto Encyclopedia of Genes and Genomes) pathway analysis through miRNA target gene‐enriched molecular pathways showed lysine degradation, axonal guidance, GABAergic synapse, and morphine addiction as pathways targeted by the significantly upregulated miRNAs. Conversely, downregulated miRNAs targeted diverse pathways like ECM‐receptor interaction, fatty acid biosynthesis, and the TGF‐β signaling pathway (Fig. 1F,G, Table S3). These findings indicate possible involvement of these pathways in cortical demyelination of progressive MS brains.

### Gene expression profiles of demyelinated cortical MS lesions

**Figure 2:**
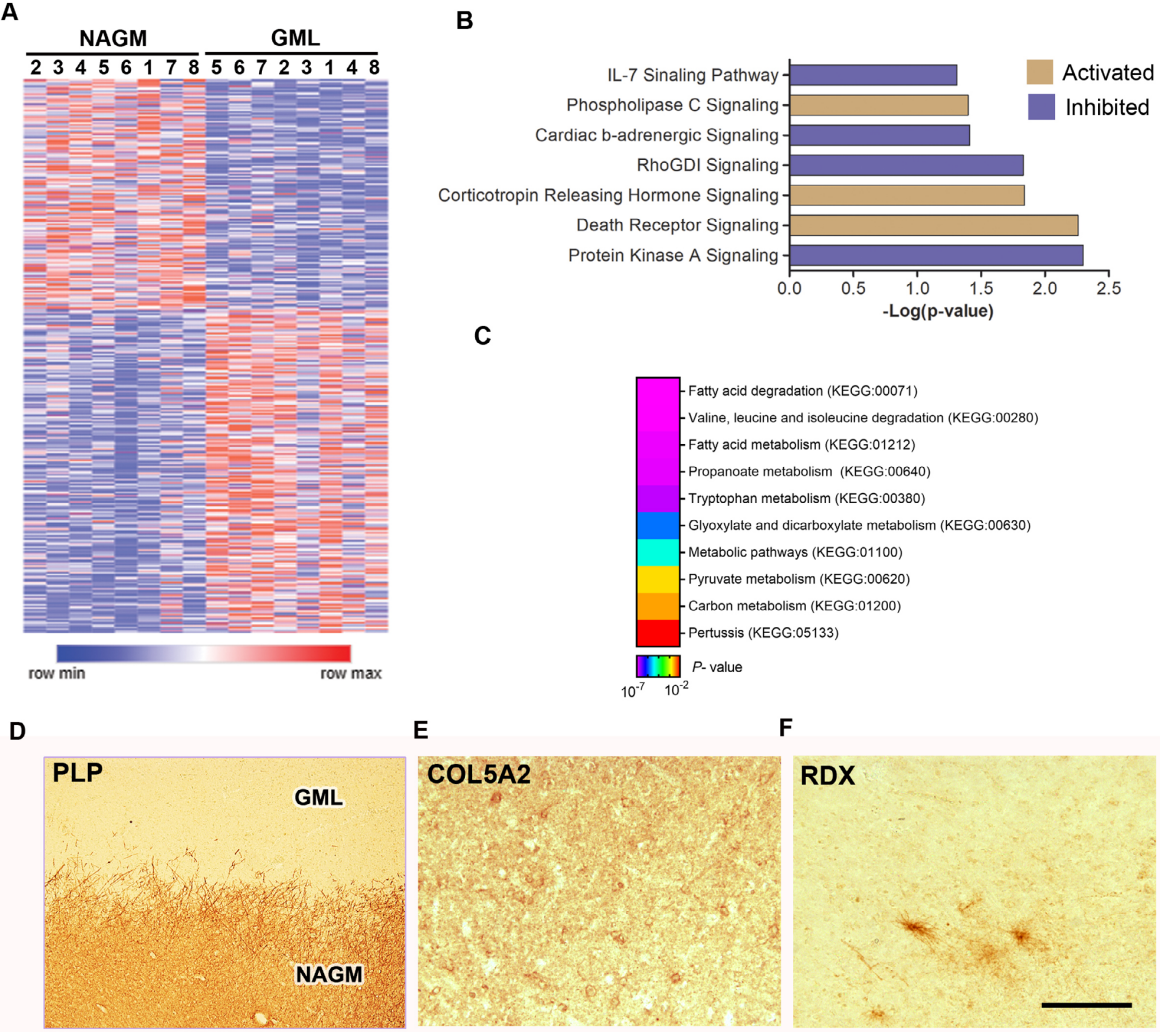
Gene expression profiling of GMLs from progressive MS brain. (A) Heatmap showing DEGs of NAGM and GML tissues from progressive MS brain (*n* = 8). (B) IPA analysis of GML DEGs (*p* < 0.05) showing enriched canonical pathway based upon *z*‐scores (orange—positive; purple—negative). (C) Representative heatmap image showing KEGG pathways associated with significantly dysregulated GML DEGs from progressive MS brains. (D–F) IHC images showing PLP, COL5A2, and RDX immunostaining in a demyelinated GML and surrounding NAGM from a progressive MS brain tissue. Scale bar—100 µm.

To study the cortical gene expression profiles of demyelinated GMLs from progressive MS brains, total RNA was isolated from eight myelinated areas (NAGM) and eight demyelinated cortical lesions (GML) and compared on Affymetrix U133A and U133B microarrays (Table S4). Among the 465 significantly differentially expressed genes (DEGs, Fig. 1B, after removing multiple probes for single gene, *p *< 0.05), 290 were upregulated and 175 genes were downregulated in demyelinated cortical lesion tissue (Fig. 2A). We also confirmed the cellular expression of the top dysregulated genes (COL5A2, FC = 1.8, *p *= 0.0154 and RDX, FC = −1.5, *p *= 0.0106) in neurons and oligodendrocytes in brain tissues from MS patients (Fig. 2D–F). We used the Ingenuity Pathway Analysis (IPA) tool to analyze biological pathways affected in demyelinated cortical MS lesions. IPA analysis showed increased activity of death receptor signaling (BCL2, FASLG, TNFRSF25, and XIAP; *p *= 0.00549), corticotrophin releasing hormone signaling (ADCY6, CNR1, CRHR2, IVL, and NPR2; *p *= 0.0146), and phospholipase C signaling (ARHGEF17, HDAC7, ITGA4, LCK, and MYL4; *p *= 0.0394), and similarly inhibition of protein kinase A signaling (CDC148, PPP1R11, PTPN7, and PTPRJ; *p *= 0.00503), RhoGDI signaling (CD44, RDX; *p *= 0.0148), cardiac β‐adrenergic signaling (ADCY6 and PP1R11; *p *= 0.0392), and IL‐7 signaling (CDK2; *p *= 0.0491) (Fig. 2B). Further, functional enrichment analysis of significantly dysregulated GML genes was performed with GO‐based classification using g:Profiler, which showed association to drug metabolic processes, neurotransmitter biosynthetic processes, positive regulation of apoptotic cell clearance, and phagocytosis (Table S5) underlying the pathology of GMLs. As MS demyelinating lesions are characterized by myelin loss and remyelination failure, KEGG pathway analysis of these transcripts showed enrichment of genes related to fatty acid degradation (KEGG:00071), fatty acid metabolism (KEGG:01212), and valine, leucine, and isoleucine degradation (KEGG:0280) during cortical demyelination (Fig. 2C), highlighting ongoing myelin degradation in cortical demyelinating lesions of progressive MS brains.^24^

### IPA analysis of miRNA‐target gene signatures and pathways modulating cortical demyelinating lesions

To understand how miRNA dysregulation may be impacting gene expression in demyelinating GMLs and therefore might be associated with MS disease pathogenesis, we correlated the expression profiles of significantly dysregulated miRNAs (up/down) to their predicted/confirmed target mRNAs in GMLs using the IPA tool. The analysis showed that out of 10 significantly upregulated miRNAs, hsa‐miR‐1180, hsa‐miR‐1275, hsa‐miR‐129‐5p, hsa‐miR‐149*, hsa‐miR‐423‐5p, hsa‐miR‐433, hsa‐miR‐638, and hsa‐miR‐92b were regulating 76 significantly downregulated genes in GMLs (Table S6). IPA core analysis of targeted genes showed enrichment of Sphingosine‐1‐phosphate signaling (ASAP2B and CASP14; *p *= 0.00744), protein kinase A signaling (CDC14B, DUSP16, and PTPRJ, *p *= 0.0116), and ceramide biosynthesis (SPTLC1; *p *= 0.0202) pathways in GMLs. Similarly, 17 downregulated miRNAs were found to be targeting 73 significantly upregulated genes in GMLs (Table S7), which were associated with chondroitin sulfate degradation (HYAL3 and HYAL4; *p *= 0.00136), axonal guidance signaling (ADAM11, ITGA4, EPHA7, WNT4, and PAPPA; *p *= 0.00142), and dermatan sulfate degradation (HYAL3; *p *= 0.00154) pathways in GMLs, similar to independent miRNA targeted KEGG pathway analysis (Fig. 1F,G). Based on the comparative profiling, these results highlight the loss of miRNA regulation on target gene expression and associated pathways in demyelinated cortical lesion area in MS brain.

### Identifying brain atrophy‐associated miRNAs in cortical demyelinated lesions of progressive MS brains

Regev et al^10^ reported 87 miRNAs having significant relationships (Spearman coefficient correlation *p* < 0.05) between serum miRNAs and cortical gray matter volume (cGMV) (Table S8). As there can be significant differences between the miRNAs detected in peripheral circulation versus cortical GMLs, we compared our current data (82 miRNAs) to the study by Regev et al^10^ and found 13 miRNAs common between the two studies (Table 3). Of these 13, 4 miRNAs were found to be significantly (*p *< 0.05) dysregulated in GMLs of progressive MS brain in our study. Interestingly, these miRNAs have also been found to be present in the peripheral circulation of MS patients, mainly expressed by immune cells.^25^ DIANA‐miRPath (v.3) analysis of the four atrophy associated miRNAs predicted to regulate genes associated with diverse pathways like ECM‐receptor interaction, lysine degradation, and focal adhesion pathways (Table S9). Recent studies have highlighted importance of extra cellular matrix in CNS development and various neurodegenerative diseases including MS.^26^, ^27^

**Table 3:** Brain atrophy associated miRNAs detected in serum and gray matter lesions (miRNAs highlighted in red and italics were also validated with RT‐qPCR).

miRs#	miRNAs	GML/NAGM	*p* value
1	hsa‐miR‐106b	1.00	0.994
2	hsa‐miR‐132	1.01	0.867
3	hsa‐miR‐149*	*1.71*	*0.007*
4	hsa‐miR‐15b	0.97	0.811
5	hsa‐miR‐185	1.21	0.064
6	hsa‐miR‐195	0.96	0.717
7	hsa‐miR‐204	0.87	0.610
8	*hsa‐miR‐20a*	*0.55*	*0.002*
9	hsa‐miR‐25	0.85	0.008
10	*hsa‐miR‐29c*	*0.45*	*0.043*
11	hsa‐miR‐30e	0.64	0.107
12	hsa‐miR‐320b	0.88	0.203
13	hsa‐miR‐7	1.21	0.620

**Figure 3:**
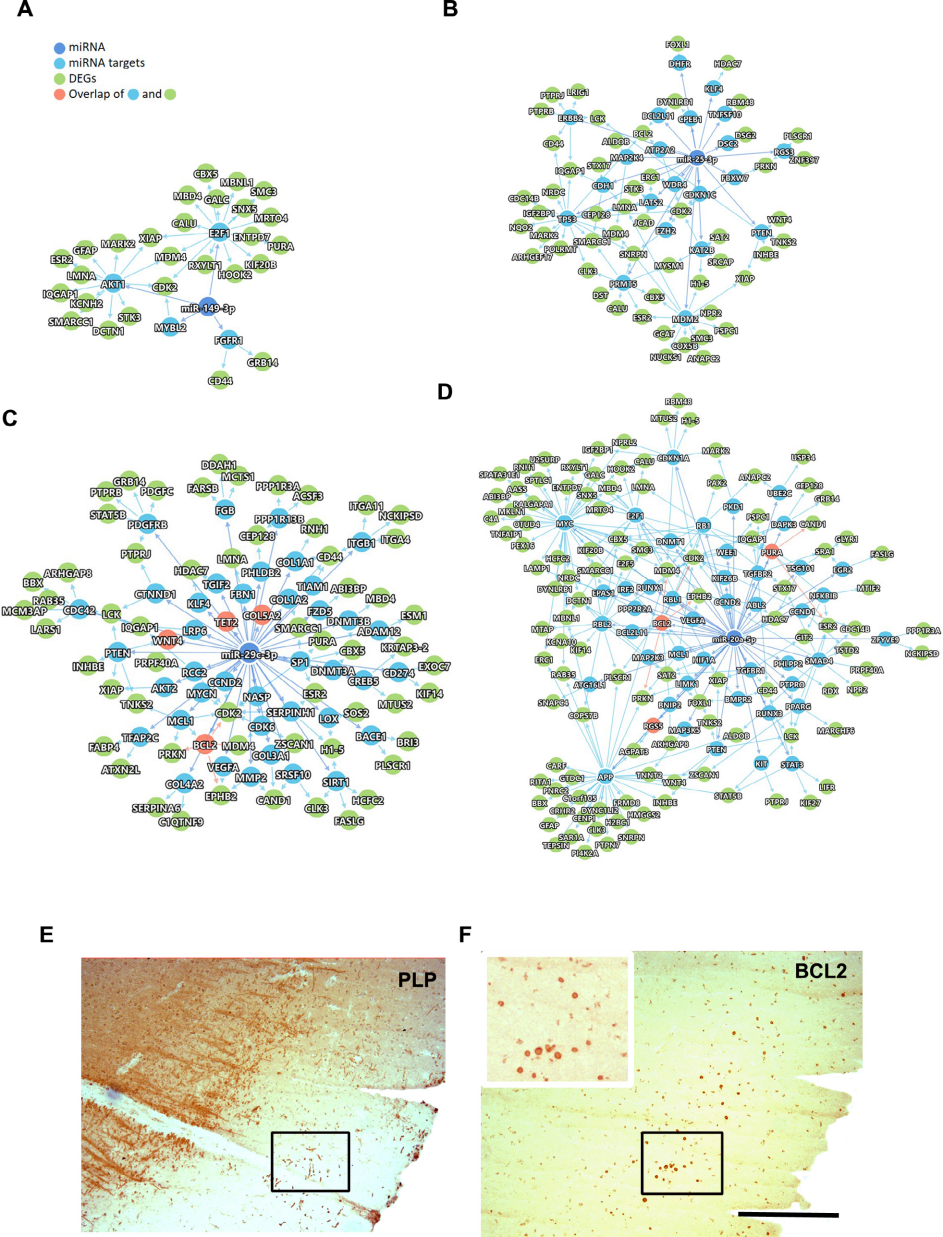
The protein–protein interactions of atrophy‐associated miRNAs (blue) and predicted/confirmed targets genes (blue/green/orange). (A) miR‐149*, (B) miR‐25, (C) hsa‐miR‐29c, and (D) hsa‐miR‐20a and the DEGs from the list of GML gene expression data. Direct miRNA targets are shown in blue circles. Proteins in orange circles represent both DEGs and miRNA direct targets. (E, F) Representative IHC images of PLP and BCL2 immunostaining in a demyelinated GML and surrounding NAGM from a progressive MS brain. Left upper boxed area highlights selected region showing increased BCL2‐positive cells in GML. Scale bar—100 µm.

We further analyzed the relationships between the four atrophy‐associated miRNAs (miR149*, miR‐20a, miR‐25a, and miR‐29c) and significant DEGs in the GMLs in the context of the human protein interactome. Using a human interactome database that contains 351,444 PPIs (among 17,706 proteins) and a functional miRNA‐target interactome of 8157 interactions (edges) among 735 miRNAs and 2766 target genes, we extracted all of the PPIs of the selected miRNA targets and the DEGs. The results indicate extensive PPIs among the miRNA targets (blue nodes) as well as the DEGs (green nodes) of all four miRNAs (dark blue nodes) (Fig. 3A–D). Interestingly, for hsa‐miR‐20a (−5p) and hsa‐miR‐29c (−3p), a few DEGs were direct targets of the respective miRNAs. For example, BCL2, RGS5, and PURA were directly targeted by hsa‐miR‐20a, whereas WNT4, TET2, COL5A2, and BCL2 were directly targeted by hsa‐miR‐29c. Indeed, many of these genes (BCL2, TET2, and COL5A2) have been indeed found to be dysregulated in biological samples from MS patients.^14^, ^28^, ^29^, ^30^ We also performed validation using immunohistochemistry to detect the presence of BCL2‐positive cells within regions of GMLs (Fig. 3E,F). These observations establish that the four miRNAs in GMLs could potentially affect the expression of the DEGs through direct as well as indirect interactions.

## Discussion

Recent reports suggest that the presence of cortical demyelination correlates with progressive neurological decline in MS patients.^31^, ^32^ As cortical lesions are difficult to detect by conventional MRI techniques, identifying markers to predict cortical involvement in MS cases would be helpful to predict the cortical component of MS. In the present study, we profiled miRNAs expression in GMLs from progressive MS brains and identified 82 miRNAs that belonged to biological pathways like ECM‐receptor interaction, axon guidance, and adherens junction signaling. The target mRNAs of the identified miRNAs showed enrichment of pathways like sphingosine‐1‐phosphate signaling, chondroitin sulfate degradation, and axonal guidance. Finally, we identified miRNAs that are changed in GMLs and correlate with MRI measurements of cGMV.^10^ Taken together, this study identified a list of miRNAs and biological pathways that are changed in GMLs from progressive MS brain tissues and correlate with the cGMV.

Several studies have been performed to analyze miRNA expression in different biological samples (serum/plasma, cerebrospinal fluid, and immune cells) from MS patients using techniques like microarray, RT‐qPCR, small RNA sequencing, and ISH.^33^, ^34^, ^35^, ^36^ However, there are limited studies that provide miRNA profiling data from brain tissues of MS patients. While the majority of these reports have focused on WMLs,^6^, ^9^ a recent study analyzed miRNA expression from MS brain cortical lesions and presented 20 upregulated and 11 downregulated miRNAs.^11^ Our study in comparison to this report had only two miRNAs that were common (miR‐1180 and miR‐219‐2‐3p). The differences between these two studies may be attributed to the platform used to profile miRNAs (Nanostring technology^TM^ vs. microarray platform) and the use of only control tissue for comparison with GMLs from MS brains. Interestingly, several of the significantly dysregulated miRNAs (miR‐1275, miR‐129‐5p, miR‐26a, miR‐23b, miR‐16, and miR‐338‐5p) reported in this study were common with our previous report of miRNAs identified in MS brain WMLs.^9^ The similarity of the previously published WML dataset to the GML miRNA profile suggests possible involvement of these miRNAs regulating common pathways in GMLs and WMLs from MS brains. In line with this possibility, miR‐338 has been found to play a major role during CNS myelination and myelin repair,^37^ while miR‐23b has been reported to play a critical role in suppressing leucocyte migration and halting the progression of experimental autoimmune encephalomyelitis (EAE), an animal model of MS.^38^ Similarly, miR‐129 has been reported to control axonal regeneration via regulating insulin‐like growth factor‐1 in peripheral nerve injury.^39^ Intriguingly, three miRNAs (miR‐25, miR‐30d, and miR‐100) downregulated in GMLs were upregulated in WMLs.^9^ Among the pathways targeted by these dysregulated miRNAs, axonal guidance was found to be significantly affected, probably due to loss of regulation of target gene expression (ADAM11, ITGA4, EPHA7, WNT4, and PAPPA).^40^ Other pathways, such as ECM receptor interaction, fatty acid biosynthesis, and TGF‐β‐signaling, have been proposed to be critical targets for myelin regeneration therapy for demyelinating lesions.^41^, ^42^, ^43^

One important aspect of our study was to investigate concurrent gene expression from similar lesion tissue types as used for miRNA expression analysis. This analysis helped us to identify the pathways that might be active in GMLs. Among these pathways, death receptor signaling associated molecules like death receptors (DR) and decoy receptors (DcR) have been reported in MS WMLs.^44^ Likewise, genes relating to corticotrophin releasing hormone has been found to be associated with decreased risk for MS disease.^45^ The results also identified several pathways that may be repressed in GMLs. For example, genes related to protein kinase A (cAMP‐dependent protein kinase) signaling were found to be significantly decreased in GMLs. In the nervous system, this pathway has been found to be associated with dopamine signaling.^46^ In addition to alteration of the genes and pathways, several miRNAs in GMLs were found to target pathways like fatty acid degradation/metabolism, as well as valine, leucine, and isoleucine degradation. Of particular interest was the identification of fatty acid metabolism as a top hit, which could be a potential therapeutic target for treating MS.^42^

Cortical demyelination‐associated neuronal and axonal loss are main factors connected with neurological dysfunction reported from MS patients.^47^ Previously, miRNA expression was correlated with GM atrophy measures.^10^ Based upon positive/negative correlations, the authors^10^ grouped these miRNAs as protective or pathogenic. Based upon the expression levels of these miRNAs, we found that miR‐149* (−3p) was significantly upregulated (identified as protective) in GMLs and found to be associated with genes involved in T‐cell proliferation, inhibition of apoptosis by targeting PD‐1, TIM‐3, BTLA, Foxp1, IL‐2, TNF‐α, and IFN‐γ in breast cancer cells.^48^ Similarly, miR‐20a, miR‐25, and miR‐29c (pathogenic miRNAs) were significantly downregulated in GMLs. These three miRNAs have been found to target genes involved with inflammation and apoptosis activity^49^, ^50^, ^51^ and also have been found to be enriched in CNS microglia and tissue resident macrophages.^52^ Moreover, the target gene analysis through human PPI interactome analysis identifies direct (BCL2, TET2, RGS5, and COL5A2) gene targets of these miRNAs that could form the basis of future research to investigate the role of these genes in GMLs. Interestingly, BCL2, an anti‐apoptotic protein, is expressed by oligodendrocytes and has been found to be increased within demyelinating lesions compared to the peri‐plaque WM, with the highest numbers in remyelinating lesions specifically during relapsing‐remitting disease course,^30^ thus highlighting oligodendrocyte preservation or loss in MS WMLs. Conversely, BCL2 also acts as downstream product of the protective ciliary neurotrophic factor (CNTF) signaling pathway reported to be active in MS cortex.^14^

Serum miRNAs expression can also be influenced by the presence of WMLs. Although we did not find significant overlap between our findings and serum miRNAs that correlate with WMLs,^9^, ^10^ further studies are needed to confirm if cortical atrophy associated serum miRNAs could also be influenced by presence of WMLs. Nonetheless, identification of four miRNAs in cortical lesions provides preliminary evidence of participation of these miRNAs in affecting atrophy measures in MS cortical GM.

In summary, we report a new set of miRNAs associated with cortical demyelination that are detected in serum of MS patients and are associated with cortical atrophy. The target genes of these miRNAs were found to be associated with neuronal function, fatty acid synthesis, and inflammation. As we also correlated the expression of these miRNAs in serum, our results provide a method to study cortical changes in MS brains through peripheral monitoring. These results also lay the foundation for future studies to target these miRNAs and pathways in order to investigate mechanisms underlying lesion formation and neuroprotection in progressive MS.

## Author Contributions

AT and AP performed experiments and data analysis. YD and FC contributed to Human interactome network analysis. AT drafted the manuscript. RD designed and supervised the study and finalized the draft. All authors have read and approved the final manuscript.

## Conflict of Interest

The authors declare no competing interests.

## Funding Information

This work was supported by grants from NINDS (NS096148). The MS brain collection program is supported by NINDS grant R35NS097303.

## Ethics Approval and Consent to Participate

The use of human post‐mortem brain tissue was approved by Cleveland Clinic Institutional Review Board.

## Supporting information


**Supplementary File** **1**. miRNAs detected in MS Gray matter lesions (GMLs) compared to normal appearing gray matter (NAGM).

**Supplementary File** **2**. Predicted pathways targeted by identified miRNAs cortical tissue from MS brain.

**Supplementary File** **3**. Predicted pathways targeted by upregulated and down regulated miRNAs in demyelinated cortical tissue from MS brain.

**Supplementary File** **4**. Differentially expression genes (DEGs) in Gray matter lesion (GMLs) in MS brain.

**Supplementary File** **5**. gProfiler analysis of DEGs from GML.

**Supplementary File** **6**. Genes targeted by upregulated miRNAs in GML tissue.

**Supplementary File** **7**. Genes targeted by downregulated miRNAs in GML tissue.

**Supplementary File** **8**. Serum miRNAs identified by (Regev et al., 2017) correlating (p < 0.05) with cortical gray matter volume (cGMV) measurement.

**Supplementary File** **9**. Predicted pathways targeted by gray matter atrophy associated miRNAs from MS brain.

**Supplementary Table S1**. miRNAs TaqMan assays IDs used for RT‐qPCR validation study.

## Data Availability

All data generated or analyzed during this study are included in this article (and its supplementary information files).
