# Supplementary material for: Identifying miRNAs in multiple sclerosis gray matter lesions that correlate with atrophy measures
Source: Ann Clin Transl Neurol. 2021 May 12;8(6):1279–91. doi: 10.1002/acn3.51365 (PMC8164853; doi:10.1002/acn3.51365)
Supplement: Supplementary file 10 — Supplementary Table S1. miRNAs TaqMan assays IDs used for RT‐qPCR validation study. [file ACN3-8-1279-s004.docx]

**Supplementary table 1:** miRNAs TaqMan assays IDs used for RT-qPCR validation study

| **miRs#** | **miRNAs** | **Assay ID#** |
| --- | --- | --- |
| **1** | hsa-miR-1275 | Hs06633468_s1 |
| **2** | hsa-miR-149* | Hs04231523_s1 |
| **3** | hsa-miR-1180 | Hs04273417_s1 |
| **4** | hsa-miR-129-5p | Hs04231518_s1 |
| **5** | hsa-miR-20a | Hs01382425_m1 |
| **6** | hsa-miR-584 | Hs04231683_s1 |
| **7** | hsa-miR-126 | Hs04273250_s1 |
| **8** | hsa-miR-29c | Hs04231537_s1 |
